# Natural Products for the Treatment of Obesity, Metabolic Syndrome, and Type 2 Diabetes 2014

**DOI:** 10.1155/2015/392681

**Published:** 2015-03-10

**Authors:** Menaka C. Thounaojam, Srinivas Nammi, Ravirajsinh Jadeja

**Affiliations:** ^1^Department of Biochemistry and Cancer Biology, Meharry Medical College School of Medicine, Nashville, TN 37208, USA; ^2^School of Science and Health, University of Western Sydney, NSW 2751, Australia; ^3^National Institute for Complementary Medicine (NICM), University of Western Sydney, NSW 2751, Australia; ^4^Division of Gastroenterology/Hepatology, Department of Medicine, Medical College of Georgia, Georgia Regents University, Augusta, GA 30912, USA

In the modern and contemporary world, the incidence of obesity, metabolic syndrome, and type II diabetes is rising continuously due to modernization in life style and dietary habits. Herbal medicines have been shown to be beneficial against coexistence of these ailments. In 2013, we published a special issue on* “Natural products for the treatment of obesity, metabolic syndrome, and type 2 diabetes”* in Evidence-Based Complementary and Alternative Medicine (eCAM). After receiving an overwhelming number of submissions and successful compilation of research/review articles in the first season, the decision to publish annual special issue on this subject has been made by eCAM. Herein we present the 2014 issue in this series. This special issue consists of 5 original research papers and 4 review articles.

A study by S. J. Song et al. evaluated the potential of decaffeinated green coffee bean extract (DGCBE) in regulating diet-induced obesity and insulin resistance using a mouse model. The authors showed that 0.3% supplementation of DGCBE significantly reduces visceral adiposity and improved insulin resistance that can be attributed to 5-caffeoylquinic acid (CQA) and other polyphenols. The benefits of DGCBE are further attributed to a possible downregulation of the genes associated with adipogenesis and inflammation in the adipose tissue. This study suggested that decaffeinated green coffee beans can be used as a therapeutic agent against obesity and metabolic syndrome. Another study by H. M. Eid et al. reported that Lingonberry (*Vaccinium vitis-idaea* L.) intake improves blood glucose in a mouse model of diet-induced obesity without significantly affecting food intake or body weight. The proposed potential mechanism is via enhanced expression of GLUT4 in skeletal muscle and reduced hepatic steatosis. Collectively the authors put forward the potential of Lingonberry in treating insulin resistance and obesity. Similarly, M. N. Abu et al. suggested that oral administration of* Tinospora crispa* crude extract to high fat diet-fed insulin resistant rats exhibits antidiabetic, antihypercholesterolemic, and hepatoprotective effects and thus could be useful in treating obesity in patients with insulin resistance and type II diabetes.

A study by T. Park et al. reported antiadipogenic potential of Alismatis rhizome. It was shown that AOE suppresses adipocyte differentiation in OP9 cells by downregulating the expression of C/EBP and consequently decreasing PPAR and C/EBP levels. In another study, El-Houri and colleagues performed screening of bioactive metabolites in plant extracts that modulate glucose uptake and fat accumulation. The authors used various aerial and underground parts of seven different plants to screen their efficacy in reducing glucose uptake and fat accumulation using different* in vitro* experimental models. This screening study provides an ideal platform for further in-depth evaluation of these herbs in regulating insulin resistance and obesity.

The review articles in this special issue focus on the efficacy of herbal medicines in regulating diabetes, obesity, and nonalcoholic steatohepatitis. An extensive review article by L. Zhang et al. summarized the use of Chinese herbal medicines mentioned in 54 famous ancient materia medica monographs. The authors further discussed the available experimental studies on these herbs with their mechanisms of action and limitations. In another review, M. Getek and colleagues discussed the antidiabetic potential of leguminous plant components focusing on both preclinical and clinical research conducted from 2004 to 2014 and highlighted key benefits with limitations. We sincerely hope that this type of extensive review will provide a future direction to the ongoing research in the field of herbal medicines and type 2 diabetes. P.-W. Chong et al. have meticulously reviewed the efficacy and safety of Litramine (IQP-G-002AS, an* Opuntia ficus-indica* derived fiber) for weight management. This review article mentioned positive results for fecal fat excretion and weight loss and also discussed the safety aspects. In the last decade, the potential of herbal medicines in regulating nonalcoholic steatohepatitis (NASH) has received much attention. In this context, the review by Jadeja et al. extensively discussed the beneficial role of various herbal extracts, phytocompounds, and polyherbal formulations in the management of NASH.

Collectively this special issue provides meticulous compilation of our current knowledge on the role of herbal medicines in regulating obesity, metabolic syndrome, and type 2 diabetes and possible future avenues that needs to be filled. We sincerely hope that this kind of annual issue series will have a long-term impact and can gather a community around it in a short time in much the same way as a successful annual conference does.

